# Impact of Biopsy-to-Radical Prostatectomy Interval on Adverse Pathological Outcomes in High-Risk Localized and Locally Advanced Prostate Cancer

**DOI:** 10.3390/diagnostics16162662

**Published:** 2026-08-20

**Authors:** Lorand Tibor Reman, Olivér Árpád Vida, Călin Chibelean, Daniel Porav-Hodade, Ciprian Todea Moga, Veronica Maria Ghirca, Raul-Dumitru Gherasim, Rares-Florin Vascul, Orsolya-Brigitta Katona, Szabolcs André, Edva Anna Frunda, Orsolya Katalin Ilona Martha

**Affiliations:** 1 Department of Urology, George Emil Palade University of Medicine, Pharmacy, Science and Technology of Targu Mures, 540139 Targu Mures, Romania; tibor.reman@umfst.ro (L.T.R.); daniel.porav-hodade@umfst.ro (D.P.-H.); ciprian.todea@umfst.ro (C.T.M.); maria.ghirca@umfst.ro (V.M.G.); raul-dumitru.gherasim@umfst.ro (R.-D.G.); raresvascul@gmail.com (R.-F.V.); orsolya.martha@umfst.ro (O.K.I.M.); 2 Department of Urology, Mures Clinic County Hospital, Piata Bernady Gyorgy, Nr. 6, 540072 Targu Mures, Romania; szabolcs865@gmail.com; 3 Department of Anaesthesiology and Intensive Care, County Emergency Clinic Hospital of Targu Mures, 540139 Targu Mures, Romania; katona.brigics@gmail.com; 4 Institution Organizing University Doctoral Studies (I.O.S.U.D.), George Emil Palade University of Medicine, Pharmacy, Science and Technology of Targu Mures, 540139 Targu Mures, Romania; frunda.anna@gmail.com

**Keywords:** prostate cancer, biopsy to radical prostatectomy interval, positive surgical margins, extraprostatic extension, seminal vesicle involvement

## Abstract

**Background:** In high-risk prostate cancer, the optimal timing of radical prostatectomy after diagnostic biopsy remains a clinically important issue. A real-world delay between diagnosis and radical treatment frequently occurs, raising concerns about potential disease progression in high-risk patients. **Methods:** We conducted a single-center retrospective study including patients with high-risk localized and locally advanced prostate cancer who underwent radical prostatectomy from January 2016 to January 2026. Patients were stratified according to biopsy-to-radical prostatectomy interval into two groups: <90 days or ≥90 days. Adverse pathological outcomes were defined as extraprostatic extension, seminal vesicle involvement, positive surgical margins and lymph-node involvement. Univariable comparisons and multivariable logistic regression analyses were performed to identify independent predictors of adverse pathology. **Results:** A total of 158 patients with high-risk prostate cancer were included, of whom 67 (42.4%) underwent open- or laparoscopic radical prostatectomy within 90 days after biopsy and 91 (57.6%) after ≥90 days. On univariable analysis, the rates of extraprostatic extension were 59.7% vs. 63.7% in the <90-day and ≥90-day groups (*p* = 0.606), seminal vesicle involvement was observed in 22.4% vs. 24.2% (*p* = 0.627), positive surgical margins in 35.8% vs. 39.6% (*p* = 0.632), and lymph node involvement in 6% vs. 5.5% (*p* = 0.999). In multivariable logistic regression, a biopsy-to-radical prostatectomy interval ≥90 days was not independently associated with extraprostatic extension (OR 1.38, 95% CI 0.68–2.77, *p* = 0.371), seminal vesicle involvement (OR 1.43, 95% CI 0.63–3.27, *p* = 0.391) or positive surgical margins (OR 1.31, 95% CI 0.66–2.62, *p* = 0.443). The number of positive biopsy cores independently predicted extraprostatic extension (OR 1.17, 95% CI 1.04–1.32, *p* = 0.008), while higher PSA independently predicted seminal vesicle involvement (OR 1.05, 95% CI 1.01–1.10, *p* = 0.025) and positive surgical margins (OR 1.05, 95% CI 1.00–1.10, *p* = 0.027). **Conclusions:** In this real-world cohort of patients with high-risk prostate cancer undergoing radical prostatectomy, no statistically significant independent association was detected between a biopsy-to-radical prostatectomy interval ≥90 days and extraprostatic extension, seminal vesicle involvement, or positive surgical margins. However, the confidence intervals remained compatible with potentially clinically meaningful differences, and residual confounding from clinician-driven prioritisation and unmeasured preoperative factors cannot be excluded. Therefore, these findings should not be interpreted as demonstrating equivalence or the safety of delaying surgery.

## 1. Introduction

Prostate cancer (PCa) remains one of the most frequently diagnosed malignancies among men worldwide and continues to represent a major public health burden. According to recent global cancer estimates, prostate cancer accounted for more than 1.4 million new cases in 2022 and ranked among the leading cancers in men [1]. Although many prostate cancers follow an indolent course, high-risk localized and locally advanced prostate cancer represents a clinically distinct subgroup associated with a substantially higher probability of biochemical recurrence, need for adjuvant treatment, metastatic progression, and cancer-specific mortality [2]. However, the management of high-risk disease represents a continuous clinical challenge and timely definitive treatment remains a central component of disease management. In this context, treatment timing has emerged as a potentially actionable factor that may influence oncological outcomes in this subgroup. Data focusing exclusively on high-risk patients and evaluating pathological endpoints remain limited and heterogeneous.

Radical prostatectomy (RP), usually combined with extended pelvic lymph node dissection when indicated, represents an established curative-intent treatment option for selected patients with high-risk localized and locally advanced prostate cancer and may be integrated within multimodal therapeutic strategies [3]. Beyond local tumour control, radical prostatectomy provides definitive pathological staging and allows direct assessment of adverse pathological features, including extraprostatic extension, seminal vesicle involvement, positive surgical margins and lymph node involvement [4]. These findings are clinically relevant because they influence postoperative risk stratification, follow-up intensity, and the potential indication for adjuvant or salvage treatment [5].

The interval between diagnostic prostate biopsy and radical prostatectomy is an increasingly relevant issue in real-world clinical practice as it raises concerns about potential disease progression in high-risk patients [6]. Treatment delays may arise from multiple sources, including surgical waiting lists, completion of staging investigations, multidisciplinary decision-making, patient optimization, management of comorbidities, patient preference, and institutional resource limitations [7].

Treatment delay is a clinically relevant concern across multiple malignancies, although its impact varies according to tumor biology and treatment modality. In breast cancer, progressively longer intervals between diagnosis and surgery have been associated with lower overall and disease-specific survival, while in muscle-invasive bladder cancer, delaying radical cystectomy beyond 12 weeks has been associated with increased disease-specific and all-cause mortality. These findings indicate that acceptable surgical waiting intervals cannot be generalized across malignancies and emphasize the need for tumor- and risk-specific evaluation of treatment timing [8,9,10].

The impact of this surgical delay on pathological and oncological outcomes in high-risk localized and locally advanced PCa remains a highly controversial topic in the recent literature. Some contemporary systematic reviews suggest that definitive treatment for high-risk PCa can generally be postponed for up to approximately three months without clear oncological compromise, whereas longer delays, particularly beyond six to nine months, may be associated with worse biochemical or pathological outcomes [11]. Other studies over the last five years suggest that deferring curative surgical treatment, particularly beyond the 3-month (90 days) threshold, may be associated with an increased incidence of adverse pathological outcomes [12]. Other reviews have emphasized that longer delays may be more problematic in high-risk disease, highlighting the need for additional real-world data focused specifically on high-risk cohorts [13].

Given these uncertainties, a rigorous evaluation of the wait-time effect becomes essential for optimizing therapeutic workflow exclusively in high-risk localized and locally advanced prostate cancer. This subgroup is biologically aggressive, more likely to harbour adverse pathological features at surgery, and more likely to require multimodal treatment. Therefore, distinguishing the effect of surgical delay from the effect of baseline tumour biology is essential.

Despite growing interest in treatment timing, real-world evidence specifically addressing high-risk localized and locally advanced prostate cancer remains limited and inconsistent. The present study aimed to assess whether a biopsy-to-radical prostatectomy interval ≥90 days is independently associated with adverse pathological outcomes, such as extraprostatic extension, seminal vesicle involvement, positive surgical margins or lymph node involvement in patients with high-risk prostate cancer undergoing radical prostatectomy.

## 2. Materials and Methods

A single-center, retrospective cohort study was conducted, including patients diagnosed with high-risk localized or locally advanced prostate cancer who underwent radical prostatectomy between January 2016 and January 2026. All surgical interventions were performed via open or laparoscopic approaches at a tertiary urology center and all patients were identified from an institutional prostate cancer surgery database. Patients with incomplete clinical or pathological data for the primary variables of interest were excluded. Patients who received neoadjuvant systemic or local treatment before radical prostatectomy were also excluded, in order to minimize treatment-related confounding of final pathological findings. Given the exploratory nature of the study, no formal sample size calculation was performed.

The study methodology, including the eligibility and risk-stratification criteria, was established in 2025, before completion of data collection and statistical analysis. Consequently, patients were classified according to the 2025 European Association of Urology (EAU) Guidelines on Prostate Cancer, which represented the applicable guideline version at the time the study methodology was developed [14]. Locally advanced prostate cancer was defined as cT3–4 or cN+ disease. In the present cohort, all locally advanced cases were classified as cT3. Accordingly, the study cohort comprised patients with either high-risk localized or locally advanced prostate cancer. No formal institutional prioritisation protocol based on tumour aggressiveness was implemented during the study period. Research-specific data extraction and statistical analysis were performed after ethics committee approval.

Only patients fulfilling high-risk localized and locally advanced prostate cancer criteria were included. High-risk disease was defined according to established clinicopathological criteria, including at least one of the following: preoperative prostate-specific antigen (PSA) > 20 ng/mL or biopsy International Society of Urological Pathology (ISUP) grade group 4/5, and/or clinical T2c (cT2c) stage disease or locally advanced disease (cT3). A proportion of patients fulfilled high-risk criteria based on clinical staging or PSA level despite lower biopsy ISUP grade. No formal prioritization protocol based on tumour aggressiveness was implemented.

Data were systematically extracted from institutional electronic medical records. Preoperative and demographic variables, prostate biopsy parameters and perioperative data were collected. The main exposure variable was the interval between diagnostic prostate biopsy and radical prostatectomy, calculated in days. For the primary analysis, patients were stratified into two groups according to biopsy-to-surgery interval: <90 days and ≥90 days. The 90-day threshold was selected based on its clinical relevance and previous literature evaluating the potential impact of treatment delay in high-risk localized and locally advanced prostate cancer. In addition to the predefined categorical analysis, the biopsy-to-radical prostatectomy interval was described as a continuous variable and reported as median and interquartile range. Longer waiting times were additionally categorized as 90–120, 121–180, and >180 days to provide a more detailed description of the delay distribution.

Preoperative clinical T stage was determined by digital rectal examination, rather than imaging, in accordance with the EAU classification system, and was categorized as cT2b, cT2c, or cT3. Patients with cT2b disease were included only when they fulfilled another EAU high-risk criterion, namely PSA > 20 ng/mL and/or biopsy ISUP grade group 4–5. Further subdivision of cT3 disease was not consistently available; therefore, cT3a and cT3b were analyzed as a combined category. Although the 2026 EAU Guideline update became available during manuscript revision, it was not applied retrospectively to cohort eligibility because the study methodology and risk-classification criteria had been prespecified in 2025. The 2026 Guidelines were instead used to contextualize contemporary management in Section 4.

All patients underwent radical prostatectomy using either an open or laparoscopic approach, according to surgeon preference, patient characteristics, and institutional practice at the time of surgery. Extended pelvic lymph node dissection was performed according to oncological indication, preoperative nomograms, and institutional protocol for high-risk prostate cancer. Final pathological assessment was performed on radical prostatectomy specimens according to standard pathological procedures. The following adverse pathological outcomes were evaluated: extraprostatic extension, seminal vesicle involvement, positive surgical margins and lymph node involvement. All pathological specimens were evaluated by a dedicated genitourinary pathologist according to standardized criteria. Throughout the study period, both open and laparoscopic radical prostatectomy were performed contemporaneously by the same group of urological surgeons. Prostate biopsy methodology remained consistent, with all biopsies performed using a systematic transrectal ultrasound-guided technique according to the same institutional protocol. Pelvic lymph-node dissection was performed in all patients, with the anatomical extent determined according to oncological indication, preoperative nomograms, and institutional protocol. Pathological nodal status was available for the entire cohort and was classified as pN0 in the absence of nodal metastases and pN1 when at least one metastatic regional lymph node was identified.

The primary objective was to determine whether a biopsy-to-radical prostatectomy interval ≥90 days was independently associated with adverse pathological outcomes. Secondary analyses evaluated whether baseline preoperative tumour characteristics were associated with adverse pathological findings.

All statistical analyses were performed using SPSS software (IBM SPSS Statistics for Windows, version 32 (IBM Corp., Armonk, NY, USA)). Descriptive statistics were generated for the entire cohort and stratified by the surgical delay interval (<90 days vs. ≥90 days). Variables were selected based on clinical relevance and prior evidence. Continuous variables were explored for data distribution and reported as median with interquartile range (IQR), while categorical variables were reported as absolute frequencies and percentages. Univariable comparisons between the <90-day and ≥90-day groups were performed using the Mann–Whitney U test for continuous variables and Pearson’s chi-square test or Fisher’s exact test for categorical variables, as appropriate. Multivariable binary logistic regression models were constructed to evaluate the independent association between biopsy-to-radical prostatectomy interval ≥90 days and each adverse pathological outcome. Separate models were built for extraprostatic extension (EPE), seminal vesicle involvement (SVI) and positive surgical margins (PSM). Because lymph-node involvement occurred in only nine patients, the number of events was insufficient to support a reliable multivariable model with the prespecified covariates. Covariates included clinically relevant preoperative variables, such as age at surgery, PSA, total number of biopsy cores, number of positive biopsy cores, and biopsy ISUP grade group. Results were reported as odds ratio (OR) with 95% confidence intervals (CI). Multicollinearity among covariates was assessed prior to model inclusion. A two-sided *p*-value of <0.05 was considered statistically significant. Given the limited number of events for certain outcomes, multivariable models should be interpreted with caution.

## 3. Results

A total of 158 (100%) patients with high-risk localized and locally advanced prostate cancer who underwent radical prostatectomy were included in the final analysis. The cohort was stratified according to the biopsy-to-radical prostatectomy interval using a clinically relevant 90-day threshold. 67 (42.4%) underwent radical prostatectomy within 90 days after prostate biopsy, whereas 91 (57.6%) patients underwent surgery after an interval of ≥90 days. The <90-day group was considered the reference group for subsequent comparisons and multivariable analyses.

The median biopsy-to-radical prostatectomy interval was 96.5 days (IQR 75–125.5; range 29–291) in the overall cohort. The median interval was 70 days (IQR 57–82; range 29–89) in the <90-day group and 118 days (IQR 100–159; range 91–291) in the ≥90-day group. Among the 91 patients who underwent surgery after ≥90 days, 47 (51.6%) were operated between 90 and 120 days, 29 (31.9%) between 121 and 180 days, and 15 (16.5%) after more than 180 days.

No statistically significant differences were identified in the measured baseline variables between the two treatment-delay groups. The median age at the time of radical prostatectomy was 65 years (IQR: 63–70) for patients in the <90-day group, compared to 68 years (IQR: 64–70) for those in the ≥90-day group. Median BMI was 26.35 kg/m^2^ (IQR 24.63–29.23) in the <90-day group and 27.13 kg/m^2^ (IQR 24.85–29.41) in the ≥90-day group. Regarding perioperative parameters, the median operative time was recorded at 250 min (IQR: 200–300) in the <90-day group and 255 min (IQR: 210–280) in the delayed group. Postoperative length of stay in the Intensive Care Unit (ICU) was 3 days (IQR 2–4) in both treatment-delay groups.

Regarding biopsy-related variables, the number of biopsy cores did not differ significantly between the <90-day and ≥90-day groups: 12 (IQR: 12–14) vs. 12 (IQR: 12–14), *p* = 0.814. No statistically significant difference was identified in the number of positive biopsy cores between the groups: 6 (IQR 4–9) in the <90-day group versus 6 (IQR 4–8) in the ≥90-day group (*p* = 0.488). Median PSA was numerically higher among patients who underwent radical prostatectomy within 90 days than among those treated after ≥90 days (13.00 versus 10.30 ng/mL, *p* = 0.097). Although this difference did not reach conventional statistical significance, its direction may reflect clinical prioritization of patients perceived as having a greater preoperative oncological risk. No statistically significant difference in biopsy ISUP grade group distribution was identified between the groups (*p* = 0.960). In the overall cohort, biopsy ISUP grade group 2 was the most frequent category, observed in 97 patients (61.4%), followed by ISUP grade group 1 in 22 patients (13.9%), ISUP grade group 3 in 19 patients (12.0%), ISUP grade group 4 in 13 patients (8.2%), and ISUP grade group 5 in 7 patients (4.4%). No statistically significant difference in biopsy tumour location was identified between the groups (*p* = 0.051), although left-sided disease was numerically more frequent in the <90-day group and right-sided disease was numerically more frequent in the ≥90-day group. However, bilateral involvement was the most common biopsy location in both groups. Biopsy invasion status did not differ significantly between groups (*p* = 0.243), with absent invasion being the most frequent finding in the overall cohort. Clinical stage was cT2b in 29 patients (18.4%), cT2c in 103 patients (65.2%), and cT3 in 26 patients (16.5%). In the <90-day group, 12 patients (17.9%) had cT2b, 40 (59.7%) had cT2c, and 15 (22.4%) had cT3 disease. In the ≥90-day group, the corresponding values were 17 (18.7%), 63 (69.2%), and 11 (12.1%), respectively. The distribution of clinical stage did not differ significantly between the two treatment-delay groups (*p* = 0.219). All patients with cT2b disease fulfilled the EAU high-risk criteria based on PSA > 20 ng/mL and/or biopsy ISUP grade group 4–5. Preoperative characteristics according to biopsy-to-radical prostatectomy interval are presented in Table 1. Regarding the EAU qualifying features, 23 patients (14.6%) had a PSA level > 20 ng/mL, 20 patients (12.7%) had biopsy ISUP grade group 4–5, and 129 patients (81.6%) fulfilled the clinical-stage criterion, comprising cT2c high-risk localized disease or cT3 locally advanced disease. Because individual patients could fulfil more than one eligibility criterion, these categories were not mutually exclusive.

Operative time was similar between patients treated within 90 days and those treated after ≥90 days, with median values of 250 min (IQR: 200–300) and 255 min (IQR: 210–280), respectively (*p* = 0.938). ICU stay was also comparable between groups, with a median of 3 days (IQR: 2–4) in both groups (*p* = 0.576). No significant differences were observed between the two groups regarding pathological T stage (*p* = 0.877). In the overall cohort, pT2c disease was identified in 60 patients (38.0%), pT3a in 61 patients (38.6%), and pT3b in 37 patients (23.4%). Prostate volume was similar between the <90-day and ≥90-day groups [40 cm^3^ (IQR: 26–50) vs. 37 cm^3^ (IQR: 27–57), *p* = 0.782]. Tumour volume did not differ significantly between groups [2.28 cm^3^ (IQR: 1.19–3.63) vs. 1.90 cm^3^ (IQR: 0.90–3.70), *p* = 0.805], and tumour volume percentage was also comparable [5.63% (IQR: 3.60–10.21) vs. 5.45% (IQR: 2.44–10.53), *p* = 0.620]. Lymph node status was similar between groups (*p* = 0.999). Overall, lymph node involvement was identified in 9 patients (5.7%), including 4 patients (6.0%) in the <90-day group and 5 patients (5.5%) in the ≥90-day group. Final ISUP grade group distribution did not differ significantly according to biopsy-to-surgery interval (*p* = 0.435). Final ISUP grade group 2 was the most frequent category, observed in 94 patients (59.5%) in the overall cohort. Positive surgical margins were observed in 60 patients (38.0%) overall, including 24 patients (35.8%) in the <90-day group and 36 patients (39.6%) in the ≥90-day group. This difference was not statistically significant (*p* = 0.632). Tumour laterality at prostatectomy did not show statistical significance between groups (*p* = 0.052), although bilateral disease was predominant in both groups, being observed in 146 patients (92.4%) overall. The distribution of surgical approach did not reach the conventional threshold for statistical significance (*p* = 0.078); however, open surgery was numerically more frequent in the <90-day group, whereas laparoscopic surgery was numerically more frequent in the ≥90-day group. ISUP grade group upgrading was identified in 51 patients (32.3%) overall. Upgrading occurred in 25 patients (37.3%) in the <90-day group and in 26 patients (28.6%) in the ≥90-day group, without a statistically significant between-group difference (*p* = 0.245). Pelvic lymph-node dissection was performed in all 158 patients. Overall, 149 patients (94.3%) were classified as pN0 and nine (5.7%) as pN1. Among patients with lymph node dissection, the overall median yield was 7 nodes [IQR 5–10]. Median lymph-node yield was higher in the <90-day group than in the ≥90-day group (8.5 [IQR 6–12] versus 6 [IQR 4–9]). Perioperative and pathological characteristics are summarized in Table 2.

Overall, univariable analysis showed no statistically significant differences between the <90-day and ≥90-day groups regarding the main adverse pathological outcomes, including extraprostatic extension, seminal vesicle involvement, positive surgical margins, and lymph node involvement. These findings showed no statistically significant unadjusted association between a biopsy-to-surgery interval ≥90 days and the evaluated adverse pathological outcomes. However, the absence of statistical significance should not be interpreted as evidence of complete baseline equivalence between the groups, particularly given the numerical difference in preoperative PSA.

To adjust for potential baseline confounders and precisely evaluate the independent impact of surgical timing alongside clinical and biopsy parameters, multivariable binary logistic regression models were utilized for each adverse pathological endpoint. The covariates included in each model were age at surgery, PSA, total number of biopsy cores, number of positive biopsy cores, biopsy ISUP grade group, and biopsy-to-radical prostatectomy interval. The results of the multivariable logistic regression models evaluating independent predictors of adverse pathological outcomes are presented in Table 3.

After adjustment, a biopsy-to-radical prostatectomy interval ≥90 days was not independently associated with extraprostatic extension (OR 1.38, 95% CI 0.68–2.77, *p* = 0.371). In this model, the number of positive biopsy cores remained an independent predictor of extraprostatic extension (OR 1.17, 95% CI 1.04–1.32, *p* = 0.008).

**Figure 1:**
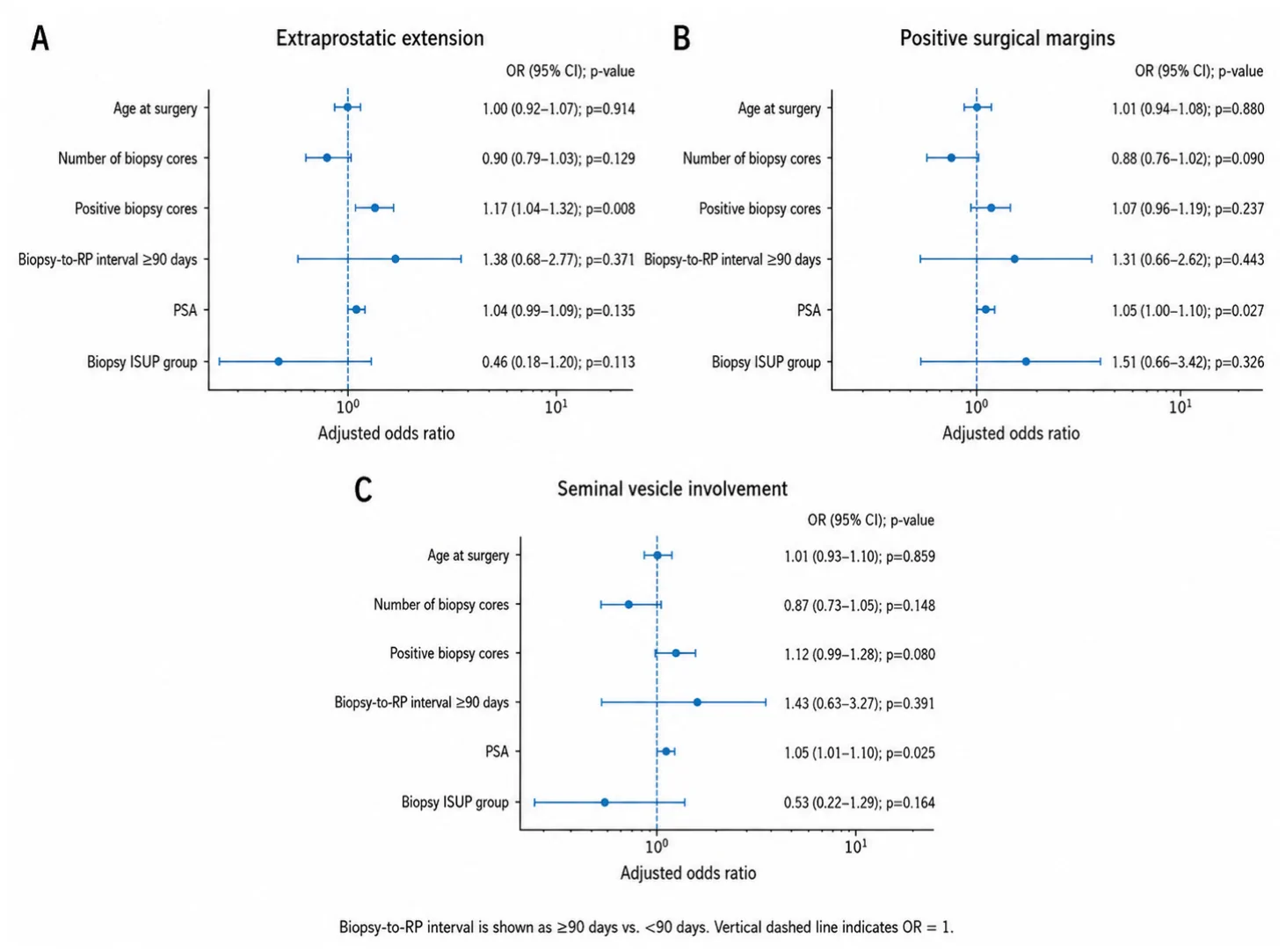
Forest plots of multivariable logistic regression models for adverse pathological outcomes. Adjusted odds ratios (ORs) with 95% confidence intervals (CIs) are presented for extraprostatic extension (**A**), positive surgical margins (**B**), and seminal vesicle involvement (**C**). Separate multivariable logistic regression models were constructed for each outcome and adjusted for age at surgery, PSA, total number of biopsy cores, number of positive biopsy cores, biopsy ISUP grade group, and biopsy-to-radical prostatectomy interval. The biopsy-to-radical prostatectomy interval is shown as ≥90 days versus <90 days. Confidence intervals crossing this line indicate no statistically significant independent association.

Similarly, surgical delay ≥90 days was not independently associated with seminal vesicle involvement (OR 1.43, 95% CI 0.63–3.27, *p* = 0.391). Higher preoperative PSA was independently associated with seminal vesicle involvement (OR 1.05, 95% CI 1.01–1.10, *p* = 0.025) (Figure 1).

For positive surgical margins, a biopsy-to-radical prostatectomy interval ≥90 days was not an independent predictor (OR 1.31, 95% CI 0.66–2.62, *p* = 0.443). In contrast, higher PSA independently predicted positive surgical margins (OR 1.05, 95% CI 1.00–1.10, *p* = 0.027).

Given the limited number of pN1 events, no multivariable logistic regression model was fitted for this outcome, and no adjusted inference regarding lymph-node involvement was attempted.

## 4. Discussion

The optimal timing of definitive surgical treatment for high-risk localized and locally advanced prostate cancer represents a critical clinical dilemma. Clinicians are often required to balance the necessity of comprehensive preoperative staging and patient optimization against the theoretical risk of rapid local or systemic disease progression. In the present study, we evaluated whether delaying radical prostatectomy for 90 days or more negatively impacts the final pathological endpoints of patients with high-risk disease. In the adjusted analyses, no statistically significant independent association was detected between a biopsy-to-radical prostatectomy interval ≥90 days and extraprostatic extension, seminal vesicle involvement, or positive surgical margins. Lymph-node involvement was analysed descriptively because the limited number of pN1 events precluded reliable multivariable modelling. These results do not permit a causal comparison between the relative contributions of baseline tumour characteristics and surgical waiting time.

The interval between biopsy and radical prostatectomy in real-world practice is not a purely biological variable. It is also influenced by administrative workflow, staging completion, multidisciplinary evaluation, patient optimization, comorbidity burden, and institutional prioritization. Therefore, surgical delay may represent a composite marker of healthcare logistics and clinical triage rather than a direct measure of tumour progression. This distinction is important when interpreting retrospective studies, because patients perceived as having more aggressive disease may be preferentially scheduled earlier, potentially attenuating or even reversing the apparent effect of delay. The 90-day cutoff used in the present study is clinically intuitive and frequently used in the literature, but it should not be interpreted as a strict biological threshold. Tumour progression is unlikely to change abruptly at 90 days. Rather, this threshold reflects a pragmatic distinction between early surgery and moderate real-world delay. Longer intervals, particularly beyond 6 to 9 months, may carry different implications and should be evaluated separately in larger cohorts [15,16].

These findings are clinically relevant because treatment timing in high-risk localized and locally advanced prostate cancer remains a frequent concern in real-world practice. Unlike low-risk prostate cancer, for which delayed treatment or active surveillance may be acceptable in selected patients, high-risk disease is biologically more aggressive and carries a higher probability of biochemical recurrence, need for adjuvant or salvage treatment, metastatic progression, and cancer-specific mortality [5,17]. Consequently, clinicians often assume that any prolongation of the interval between diagnosis and definitive treatment may translate into worse pathological findings [18]. However, prostate cancer progression is heterogeneous. In the present cohort, no statistically significant independent association was detected between an interval ≥90 days and the evaluated adverse pathological outcomes after adjustment for the measured covariates. Nevertheless, the precision of these estimates was limited, and residual confounding from clinical prioritisation cannot be excluded.

Our findings are consistent with several contemporary studies and systematic reviews evaluating the oncological safety of delayed radical prostatectomy. Nguyen et al., in a systematic review of intermediate- and high-risk prostate cancer, concluded that delays up to 3 months were not associated with worse oncological outcomes, although some studies reported higher biochemical recurrence or worse pathological outcomes with longer delays beyond 6 to 9 months [11]. Similarly, Laukhtina et al. found that a delay of approximately 3 months before radical prostatectomy appeared generally safe in intermediate- and high-risk prostate cancer, although they emphasized the low level of evidence and the heterogeneity of available studies [6,19,20]. In a large National Cancer Database analysis including more than 32,000 patients with clinically localized high-risk prostate cancer, Xia et al. reported that surgical delay up to 180 days was not associated with higher rates of pT3–4 disease, node positivity, positive surgical margins, adverse pathological score, or worse overall survival [7,21,22]. The present study adds to this literature by focusing on a real-world high-risk cohort and by evaluating several distinct pathological endpoints separately.

Nevertheless, the literature remains heterogeneous and not all studies have reached the same conclusion [23]. Berg et al. reported that delay from biopsy to radical prostatectomy may influence the rate of adverse pathological outcomes, particularly after specific time thresholds [24]. Fossati et al. also suggested that the effect of treatment delay may be more evident in high-risk patients than in lower-risk groups [25]. Zanaty et al. reported an association between surgical delay and biochemical recurrence in a Canadian cohort [12]. Conversely, Gupta et al. found that waiting up to 6 months was not associated with worse outcomes in patients with unfavorable intermediate- to very-high-risk prostate cancer [26], while Patel et al. reported that delays of up to 6 months after biopsy were not associated with grade group upgrading, extraprostatic extension, seminal vesicle invasion, positive surgical margins, or lymph node involvement [27]. Aas et al. similarly found no association between an increasing interval to radical prostatectomy up to 180 days and adverse long-term outcomes [28], and Diamand et al. reported that several months of delay did not appear to adversely affect oncological outcomes in intermediate- and high-risk patients [29,30]. These conflicting results likely reflect differences in study design, risk-group composition, delay definitions, exclusion of very long delays, use of neoadjuvant therapy, surgical prioritization practices, and endpoint selection.

An important feature of the present study is the exclusive inclusion of high-risk localized and locally advanced prostate cancer patients. Many previous analyses included mixed-risk cohorts, in which low- and intermediate-risk patients may dilute the effect of delay in biologically aggressive disease. The absence of a statistically significant association should not be interpreted as indicating that treatment timing is irrelevant. It indicates only that an independent association was not detected within the observed distribution of waiting times after adjustment for the measured covariates.

Early surgical intervention within the acute post-biopsy window is frequently complicated by periprostatic inflammation, tissue oedema, and haematomas [31]. Allowing an adequate delay—typically 6 to 8 weeks, or occasionally longer in cases of severe post-biopsy prostatitis—facilitates the resolution of these reactive changes. Operating in a non-inflamed surgical field enhances the surgeon’s ability to perform precise anatomical dissections, optimizing apical preservation and nerve-sparing techniques, which in turn may minimize the risk of iatrogenic positive surgical margins and improve postoperative functional recovery.

The association between the number of positive biopsy cores and extraprostatic extension observed in our study is biologically plausible. Positive biopsy cores reflect both tumour volume and spatial distribution within the prostate. Several studies have shown that the proportion or number of positive biopsy cores is associated with adverse pathological findings, including extraprostatic extension, positive surgical margins, seminal vesicle involvement, and biochemical recurrence [32,33]. In this context, our finding that positive biopsy cores independently predicted extraprostatic extension supports the interpretation that tumour burden at diagnosis is a key determinant of adverse pathology.

PSA also emerged as an important predictor in the present analysis. PSA remains a central component of prostate cancer risk stratification and is incorporated into major guideline-based definitions of high-risk disease [3]. Our results suggest that PSA retains prognostic information even within a high-risk population, where all patients already meet at least one adverse preoperative criterion.

Positive surgical margins, seminal vesicle involvement, extraprostatic extension, and lymph node involvement were selected as endpoints because they are clinically meaningful pathological findings after radical prostatectomy. Positive surgical margins have been associated with biochemical recurrence and, in higher-risk settings, may influence decisions regarding adjuvant or salvage treatment [34,35]. Seminal vesicle involvement and extraprostatic extension represent local disease extension beyond organ-confined cancer and are incorporated into postoperative risk stratification models. Because only nine pN1 events occurred, lymph-node involvement was reported descriptively and no adjusted inference was attempted.

The present study adds to the existing literature in several ways. First, it focuses exclusively on high-risk localized and locally advanced prostate cancer, avoiding dilution by low- and favorable intermediate-risk cases. Second, it evaluates multiple adverse pathological endpoints separately rather than relying on a composite outcome alone. Third, it adjusts for clinically relevant preoperative tumour burden markers, allowing a more balanced interpretation of the independent effect of biopsy-to-surgery interval. Finally, it reflects real-world surgical scheduling in a tertiary urology center, making the findings relevant to daily clinical practice.

Contemporary management of advanced prostate cancer is increasingly guided by molecular and genomic tumour characteristics. The 2026 EAU Guidelines recommend molecular testing in metastatic disease, including assessment of mismatch-repair deficiency and microsatellite instability. Although MSI-H/dMMR alterations are uncommon in prostate cancer, pembrolizumab may represent a biomarker-selected immune checkpoint inhibitor option for these patients; however, its tumour-agnostic indication is currently approved by the FDA but not by the EMA. Emerging immunotherapeutic strategies also include PSMA-targeted T-cell engagers. Preliminary phase 1 results with the dual-masked PSMA × CD3 T-cell engager VIR-5500 have demonstrated encouraging antitumour activity in heavily pretreated metastatic castration-resistant prostate cancer, although this treatment remains investigational and requires confirmation in larger prospective trials. These therapeutic developments emphasize the evolving systemic management of patients who progress to advanced disease but do not directly modify the surgical-timing analysis of the present localized and locally advanced cohort [36,37].

Several limitations must be acknowledged. First, this was a single-center retrospective study, and therefore selection bias cannot be excluded. Patients operated earlier may have been perceived by clinicians as having more aggressive disease, while delayed surgery may have occurred in patients with logistical, medical, or institutional constraints. Confounding by indication represents a particular concern in the present analysis. Median PSA was numerically higher in the <90-day group, suggesting that patients perceived as having a greater oncological risk may have been preferentially scheduled for earlier surgery. Although PSA was included as a continuous covariate in all multivariable models, this adjustment accounts only for measured differences and cannot completely exclude residual or unmeasured confounding. Second, the sample size was modest, particularly for less frequent endpoints such as lymph node involvement. This limits statistical power and increases the possibility of wide confidence intervals in multivariable models. Third, although the models were adjusted for clinically relevant preoperative variables, residual confounding remains possible. Important variables such as MRI findings, clinical T stage granularity, comorbidity burden, surgeon experience, surgical learning curve, and detailed lymphadenectomy template may influence pathological outcomes but were not fully captured in the analysis. Fourth, the study evaluated pathological outcomes rather than long-term oncological outcomes such as biochemical recurrence, metastasis-free survival, cancer-specific survival, or overall survival. Therefore, the absence of an association between delay and adverse pathology does not necessarily exclude a potential effect of longer delays on long-term disease control. Furthermore, only 15 patients underwent radical prostatectomy after an interval exceeding 180 days. Therefore, the present findings primarily reflect moderate real-world surgical delays and should not be extrapolated to substantially longer waiting periods.

These findings should not be interpreted as justification for unnecessary postponement of definitive treatment. The biopsy-to-surgery interval represents an administrative and clinical measure rather than a direct measure of biological progression. Pathological findings at radical prostatectomy may reflect both disease characteristics present at diagnosis and changes occurring during the waiting period; however, the present retrospective design cannot distinguish reliably between these effects. Surgical timing should therefore continue to be individualised according to guideline-based clinical assessment, while prospective multicentre studies are required to clarify the independent influence of waiting time.

An important consideration is the possibility of confounding by indication resulting from clinician-driven surgical prioritisation. Although no formal institutional protocol prioritised patients according to tumour aggressiveness, scheduling decisions may nevertheless have been influenced by the treating clinician’s perception of oncological risk. PSA was numerically higher in the <90-day group than in the ≥90-day group (13.0 vs. 10.3 ng/mL), and cT3 disease was also more frequent among patients undergoing earlier surgery (22.4% vs. 12.1%). These differences suggest that patients considered to have more aggressive disease may have been preferentially scheduled for earlier intervention. Such prioritisation could obscure or distort the observed relationship between waiting time and pathological outcomes. Although the multivariable models adjusted for several measured preoperative characteristics, they could not account for all factors potentially influencing surgical timing. MRI findings, comorbidity burden, performance status, individual clinician judgement, patient preference, and the specific organisational reasons for surgical delay were unavailable. Therefore, residual and unmeasured confounding cannot be excluded, and the absence of a statistically significant association should not be interpreted as demonstrating a causal absence of an effect of waiting time.

Although none of the adjusted associations between a biopsy-to-radical prostatectomy interval ≥90 days and the evaluated adverse pathological outcomes reached statistical significance, the estimates remained imprecise. The confidence intervals for extraprostatic extension (OR 1.38, 95% CI 0.68–2.77), seminal vesicle involvement (OR 1.43, 95% CI 0.63–3.27), and positive surgical margins (OR 1.31, 95% CI 0.66–2.62) were compatible with potentially clinically meaningful increases in odds. Accordingly, the present findings indicate that an independent association was not detected, rather than demonstrating equivalence between the two treatment-delay groups. This distinction is particularly important given the exploratory design and the absence of a formal sample-size calculation.

## 5. Conclusions

In this retrospective exploratory cohort of patients with high-risk localized or locally advanced prostate cancer, no statistically significant independent association was detected between a biopsy-to-radical prostatectomy interval ≥90 days and extraprostatic extension, seminal vesicle involvement, or positive surgical margins. However, the confidence intervals of lymph-node involvement remained compatible with potentially clinically meaningful increases in odds, and residual confounding from clinician-driven surgical prioritisation and unmeasured preoperative factors cannot be excluded. These findings should therefore not be interpreted as demonstrating equivalence between treatment-delay groups or as establishing the safety of delaying surgery. Larger, adequately powered prospective or multicentre studies are required to define more precisely the oncological implications of surgical waiting time.

## Figures and Tables

**Table 1:** Preoperative characteristics stratified by biopsy-to-radical prostatectomy interval. Values are presented as median (IQR) for continuous variables and as *n* (%) for categorical variables. *p*-values refer to comparisons between the <90-day and ≥90-day groups.

	Overall Cohort	<90 Days	≥90 Days	*p*-Value
Number of patients	158 (100%)	67 (42.4%)	91 (57.6%)	
Age (Years)	67 (63–70)	65 (63–70)	68 (64–70)	0.129
Biopsy-to-RP interval (days)	96.5 (75–125.5)	70 (57–82)	118 (100–159)	
BMI (kg/m^2^)	26.8 (24.85–29.40)	26.3 (24.6–29.2)	27.2 (24.8–29.4)	0.657
Nr. Biopsy Cores	12 (12–14)	12 (12–14)	12 (12–14)	0.814
Positive Biopsy Cores	6 (4–8)	6 (4–9)	6 (4–8)	0.488
PSA (ng/mL)	11.55 (8.27–16.09)	13.0 (8.79–17.94)	10.30 (7.6–14.37)	0.097
Biopsy ISUP grade group				0.96
1	22 (13.9%)	9 (13.4%)	13 (14.3%)	
2	97 (61.4%)	41 (61.2%)	56 (61.5%)	
3	19 (12.0%)	8 (11.9%)	11 (12.1%)	
4	13 (8.2%)	5 (7.5%)	8 (8.8%)	
5	7 (4.4%)	4 (6.0%)	3 (3.3%)	
Biopsy Tumour Location				
Right	31 (19.6%)	9 (13.4%)	22 (24.2%)	0.051
Left	34 (21.5%)	20 (29.9%)	14 (15.4%)	
Bilateral	93 (58.9%)	38 (56.7%)	55 (60.4%)	
Invasion				
Absent	125 (79.1%)	50 (74.6%)	75 (82.4%)	0.243
Perineural	26 (16.5%)	12 (17.9%)	14 (15.4%)	
Extraprostatic	7 (4.4%)	5 (7.5%)	2 (2.2%)	
cT stage				
cT2b	29 (18.4%)	12 (17.9%)	17 (18.7%)	
cT2c	103 (65.2%)	40 (59.7%)	63 (69.2%)	
cT3	26 (16.5%)	15 (22.4%)	11 (12.1%)	0.219
EAU qualifying feature				
PSA > 20 ng/mL	23 (14.6%)	10 (14.9%)	13 (14.3%)	0.910
Biopsy ISUP grade group 4–5	20 (12.7%)	9 (13.4%)	11 (12.1%)	0.802
cT2c/cT3	129 (81.6%)	55 (82.1%)	74 (81.3%)	0.902

**Table 2:** Perioperative and pathological characteristics according to biopsy-to-radical prostatectomy interval. Continuous variables are presented as median (IQR), while categorical variables are presented as *n* (%). *p*-values refer to comparisons between the <90-day and ≥90-day groups.

	Overall Cohort	<90 Days	≥90 Days	*p*-Value
Number of patients	158 (100%)	67 (42.4%)	91 (57.6%)	
Operative time (minutes)	255 (210–300)	250 (200–300)	255 (210–280)	0.938
ICU Days	3 (2–4)	3 (2–4)	3 (2–4)	0.576
Pathological T stage				
pT2c	60 (38.0%)	27 (40.3%)	33 (36.3%)	0.877
pT3a	61 (38.6%)	25 (37.3%)	36 (39.6%)	
pT3b	37 (23.4%)	15 (22.4%)	22 (24.2%)	
Prostate Volume (cm^3^)	37 (27–52.2)	40 (26–50)	37 (27–57)	0.782
Tumour Volume (cm^3^)	2.2 (1–3.7)	2.28 (1.19–3.63)	1.90 (0.90–3.70)	0.805
Tumour Volume Percentage (%)	5.46 (3.02–10.2)	5.63 (3.60–10.21)	5.45 (2.44–10.53)	0.62
Pelvic Lymph Nodes				
pN0	149 (94.3%)	63 (94.0%)	86 (94.5%)	0.999
pN1	9 (5.7%)	4 (6.0%)	5 (5.5%)	
Final ISUP grade				
1	6 (3.8%)	3 (4.5%)	3 (3.3%)	0.435
2	94 (59.5%)	34 (50.7%)	60 (65.9%)	
3	38 (24.1%)	19 (28.4%)	19 (20.9%)	
4	4 (2.5%)	2 (3.0%)	2 (2.2%)	
5	16 (10.1%)	9 (13.4%)	7 (7.7%)	
Surgical Margin Status				
Negative	98 (62.0%)	43 (64.2%)	55 (60.4%)	0.632
Positive	60 (38.0%)	24 (35.8%)	36 (39.6%)	
Tumour Laterality at Prostatectomy				
Right	6 (3.8%)	0 (0.0%)	6 (6.6%)	0.052
Left	6 (3.8%)	4 (6.0%)	2 (2.2%)	
Bilateral	146 (92.4%)	63 (94.0%)	83 (91.2%)	
Surgical Approach				
Open	82 (51.9%)	42 (62.7%)	40 (44.0%)	0.078
Laparoscopic	76 (48.1%)	25 (37.3%)	51 (56.0%)	
Extraprostatic extension	98 (62%)	40 (59.7%)	58 (63.7%)	0.606
Seminal vesicle involvement	37 (23.4%)	15 (22.4%)	22 (24.2%)	0.627
ISUP grade group upgrading				
No	107 (67.7%)	42 (62.7%)	65 (71.4%)	0.245
Yes	51 (32.3%)	25 (37.3%)	26 (28.6%)	

**Table 3:** Full multivariable binary logistic regression models for adverse pathological outcomes. Separate models were constructed for extraprostatic extension, seminal vesicle involvement, and positive surgical margins. Each model included age at surgery, PSA, total number of biopsy cores, number of positive biopsy cores, biopsy ISUP group, and biopsy-to-radical prostatectomy interval. Adjusted odds ratios for continuous variables represent the change associated with a one-unit increase. The biopsy-to-radical prostatectomy interval is presented as ≥90 days versus <90 days. OR, odds ratio; CI, confidence interval; RP, radical prostatectomy; PSA, prostate-specific antigen; ISUP, International Society of Urological Pathology.

Outcome	Predictor	Adjusted OR	95% CI	*p*-Value
Extraprostatic extension	Age at surgery	1	0.92–1.07	0.914
	Total number of biopsy cores	0.9	0.79–1.03	0.129
	Number of positive biopsy cores	1.17	1.04–1.32	0.008
	Biopsy-to-RP interval ≥90 days	1.38	0.68–2.77	0.371
	PSA	1.04	0.99–1.09	0.135
	Biopsy ISUP group	0.46	0.18–1.20	0.113
Seminal vesicle involvement	Age at surgery	1.01	0.93–1.10	0.859
	Total number of biopsy cores	0.87	0.73–1.05	0.148
	Number of positive biopsy cores	1.12	0.99–1.28	0.08
	Biopsy-to-RP interval ≥90 days	1.43	0.63–3.27	0.391
	PSA	1.05	1.01–1.10	0.025
	Biopsy ISUP group	0.53	0.22–1.29	0.164
Positive surgical margins	Age at surgery	1.01	0.94–1.08	0.88
	Total number of biopsy cores	0.88	0.76–1.02	0.09
	Number of positive biopsy cores	1.07	0.96–1.19	0.237
	Biopsy-to-RP interval ≥90 days	1.31	0.66–2.62	0.443
	PSA	1.05	1.00–1.10	0.027
	Biopsy ISUP group	1.51	0.66–3.42	0.326

## Data Availability

The data supporting the findings of this study are available from the corresponding author upon reasonable request. The data are not publicly available due to privacy and ethical restrictions related to patient confidentiality.
